# Gynecologic complications of disease-modifying therapy in women with multiple sclerosis

**DOI:** 10.1177/13524585261475100

**Published:** 2026-08-13

**Authors:** Alexandra Balshi, John P Dempsey, Yanli Zhang-James, Riley Bove

**Affiliations:** Department of Neurology, Beth Israel Deaconess Medical Center, Harvard Medical School, Boston, MA, USA; Norton College of Medicine, SUNY Upstate Medical University, Syracuse, NY, USA; Norton College of Medicine, SUNY Upstate Medical University, Syracuse, NY, USA; UCSF Weill Institute for Neurosciences, University of California San Francisco, San Francisco, CA, USA

**Keywords:** Disease-modifying therapies, multiple sclerosis

## Abstract

**Background/Objectives:**

The long-term implications of multiple sclerosis (MS) disease-modifying therapies (DMTs) on gynecologic health remain understudied. We leverage a multicenter database to evaluate whether DMTs are associated with such complications.

**Methods:**

We propensity-score 1:1 matched women with MS on (1) any versus no or (2) moderate- to high- (vs low-) immunosuppressive DMT by age, marital status, race, ethnicity, tobacco use, body mass index, human papilloma virus (HPV) vaccination, and gynecologic examinations/screenings. We then compared matched cohorts with risk ratios (RRs) and 95% confidence intervals (CIs).

**Results:**

After matching, we analyzed 115,522 women with MS. The DMT (vs no DMT) group had increased risks of HPV positivity (RR: 1.52, 95% CI: 1.35–172), cervical dysplasia (RR: 1.40, 95% CI: 1.31–1.49), cervical cancer (RR: 1.23, 95% CI: 1.03–1.46), cervicitis (RR: 1.25, 95% CI: 1.10–1.41), herpes simplex virus positivity (RR: 1.29, 95% CI: 1.14–1.45), vulvovaginitis (RR: 1.30, 95% CI: 1.23–1.37), and Bartholin cysts/abscesses (RR: 1.37, 95% CI: 1.07–1.76). Moderate- to high- (vs low-) immunosuppressive.

**Discussion:**

MS DMTs, especially more immunosuppressive ones, are associated with increased risks of several gynecologic complications. Clinicians should recognize these potential sequelae of DMTs and integrate gynecological counseling/screening into MS care.

## Introduction

The advent and widespread use of disease-modifying therapies (DMTs) has drastically improved multiple sclerosis (MS) outcomes. While these immunomodulatory medications can increase infection susceptibility,^1^ the long-term implications of DMT use on gynecologic health remain understudied even though MS disproportionately affects women.

Furthermore, though virtually all cervical cancer stems from persistent infection with human papillomavirus (HPV), and immunosuppression impairs HPV clearance,^2–4^ the relationship between MS DMTs and cervical pathology risk requires further investigation. While a French registry study identified increased cervical cancer risk in women with MS compared to controls, this study did not account for DMT use, and a similar Swedish registry study found no increased risk.^5,6^ An Australian study of 248 women with MS reported increased cervical pathology risk in those treated with high-efficacy DMTs,^7^ and other pharmacovigilance studies found similar safety signals.^8,9^ In addition, case series have reported associations with MS DMTs and vulvovaginitis,^10,11^ highlighting the need for larger, comparative studies to more definitively characterize the gynecologic risks of DMT exposure.^12^

This study leveraged a large, multicenter database to evaluate whether MS DMTs are associated with gynecologic complications: HPV positivity, cervical dysplasia and cancer, cervicitis, herpes simplex virus (HSV) positivity, vulvovaginitis, and Bartholin cysts/abscesses.

## Methods

### Data source

We utilized electronic health records (EHRs) from 105 healthcare organizations using TriNetX, a federated research network that aggregates de-identified EHRs, pharmacy records, and administrative billing systems from contributing institutions (https://trinetx.com). Most of these institutions are large academic medical centers in the United States, and TriNetX accounts for more than 20% of the US population.^13,14^ However, the network also contains data from 20 other countries (in the Americas, Europe, the Middle East, Africa, and Asia).^13,14^ Unlike insurance claims databases, TriNetX is derived from EHRs and thus includes insured and uninsured patients. Race and ethnicity data in TriNetX are derived from self-reports in the contributing EHRs. Numerous comparative studies have been successfully conducted using TriNetX.^15–20^

We established cohorts and selected outcomes for this study using codes assigned during clinical encounters for documentation and/or billing. Supplementary Table 1 presents exact codes utilized.

### Cohort design

We first identified women with an MS diagnosis code recorded between 1 January 2010 and 31 December 2025. Within this population, we created two cohorts: (1) women treated with any DMT (ocrelizumab, ofatumumab, rituximab, ublituximab, natalizumab, alemtuzumab, fingolimod, ozanimod, siponimod, ponesimod, dimethyl or diroximel or monomethyl fumarate, cladribine, teriflunomide, glatiramer, and interferons) and (2) those never exposed to DMT. We included women aged 21–65, the recommended ages for cervical cancer screening in the United States.^21^ For both groups, we set the index date as the first post-diagnosis visit during this age range (1) to reduce surveillance bias related to treatment start and (2) to avoid misclassification among individuals who initiated a DMT outside our age window.

Each cohort excluded individuals with human immunodeficiency virus, transplant recipients, or a family history of cervical, uterine, endometrial, or vaginal cancer (to reduce potential detection bias related to increased desire for screening). To reduce bias from differential loss to follow-up, we only included individuals with at least three healthcare visits to capture those actively engaged with the healthcare system.

### Outcomes

Outcomes were binary indicators of incidence of HPV positivity, cervical dysplasia and cancer, cervicitis, HSV positivity, vulvovaginitis, and Bartholin cysts/abscesses occuring after the index date. Outcomes were ascertained using International Classification of Diseases diagnosis codes or laboratory/pathology data, including swab-based testing for HPV and HSV, culture-based testing for cervicitis, cervical cytology for dysplasia, and cervical cancer identified through cytology and linkage to oncology databases. All codes are listed in Supplementary Table 1.

### Statistical analyses

All analyses were performed on the TriNetX online platform. To reduce potential confounding, propensity score matching (PSM) was performed between cohorts using a 1:1 greedy nearest-neighbor matching algorithm without replacement, with a caliper of 0.1 standard deviations of the logit of the propensity score. We balanced characteristics known to affect gynecologic pathology risk:^21^ age, marital status, race, ethnicity, tobacco use, body mass index (BMI), HPV vaccination, cervical cancer screening (ever vs never), gynecological examinations (ever vs never), and socioeconomic status indicators (using methods established by prior TriNetX studies).^22–24^ Screening frequency was not modeled, as this should increase following abnormal findings and thus introduce post-outcome adjustment bias. Our vulvovaginitis comparisons also matched by corticosteroid/antibiotic use and hemoglobin A1c.^25^

During PSM, if a patient’s record does not include a specific diagnostic/clinical code, TriNetX assumes they have not had that condition/procedure; uncoded variables are treated as absent rather than missing. All covariates were measured at the index date using an unrestricted lookback period (any time prior to index). Balance after matching was assessed using standardized mean differences (SMDs), with SMDs < 0.1 indicating adequate balance. After matching, we calculated risk ratios (RRs), 95% confidence intervals (CIs), and *p*-values from the matched cohorts without additional adjustment. CIs were estimated using standard normal approximation methods. Individuals with cervical cancer diagnosed prior to the index date were excluded from cervical cancer analyses only, given the comparatively low recurrence of cervical cancer relative to other outcomes evaluated.

We then conducted a sensitivity analysis for our primary DMT versus no DMT comparison, restricting both cohorts to individuals with ⩾3 MS codes within a calendar year.^26^ In this analysis, healthcare engagement was additionally included in the PSM (Supplementary Table 2).

We also conducted pre-specified subgroup analyses stratified by relative magnitude of immunosuppression, categorizing DMTs as lower- (glatiramer acetate, interferons, and teriflunomide), moderate- (natalizumab, sphingosine-1-phosphate receptor modulators, fumarates, cladribine, alemtuzumab), and higher-immunosuppression (anti-CD20 therapies). We present rationale for these categories in Supplementary Table 3. For these subgroup analyses, PSM was performed independently for each pairwise comparison using the original unmatched cohorts, with identical covariate definitions and matching parameters as in the primary analysis (Supplementary Tables 4–7). To prevent exposure overlap, each group excluded women with any history of a DMT in one of the other groups. We also combined the moderate- and high-immunosuppression groups and compared them to the low-immunosuppression group. Multiple comparisons were addressed using the Benjamini–Hochberg false discovery rate procedure with a threshold of 0.05.

### Standard protocol approvals, registrations, and patient consents

To ensure patient confidentiality, all data sets within TriNetX are fully de-identified in compliance with the Health Insurance Portability and Accountability Act and the General Data Protection Regulation. The deidentification process involves the removal of personal identifiers such as names, addresses, and any other direct patient identifiers, with data aggregated at the cohort level to prevent re-identification. This study followed STROBE guidelines.

## Results

From an initial cohort of 172,627 women with MS identified (62,227 on any DMT, 110,400 on no DMT), PSM yielded two cohorts of 57,761 individuals each (115,522 total) that were well-balanced for baseline characteristics (Table 1) including mean (± standard deviation) age at index (43.9 ± 11.3 vs 43.9 ± 11.5 years). The DMT group had a numerically longer mean follow-up time (6.84 ± 4.22 vs 5.45 ± 4.32 years) than the non-DMT group but spread was similar (interquartile ranges 6.85 vs 7.07 years).

**Table 1.** Baseline characteristics of women with multiple sclerosis on versus off disease-modifying therapy before and after propensity score matching.

	Before PSM			After PSM		
	On DMT (*n* = 62,227) Mean ± SD or *n* (%)	Off DMT (*n* = 110,400) Mean ± SD or *n* (%)	SMD	On DMT (*n* = 57,761) Mean ± SD or *n* (%)	Off DMT (*n* = 57,761) Mean ± SD or *n* (%)	SMD
Age at index date	43.2 ± 11.4	46.8 ± 11.6	0.3093	43.9 ± 11.3	43.9 ± 11.5	0.0022
*Race*						
Asian	578 (0.93)	1512 (1.37)	0.0414	572 (0.99)	563 (0.98)	0.0016
Black or African American	9647 (15.50)	15,452 (14.0)	0.0425	8676 (15.02)	8455 (14.64)	0.0108
Native American or Alaska Native	239 (0.38)	482 (0.44)	0.0082	224 (0.39)	183 (0.32)	0.0120
Native Hawaiian or Other Pacific Islander	60 (0.10)	158 (0.14)	0.0135	60 (0.10)	47 (0.08)	0.0074
White	44,548 (71.59)	77,777 (70.45)	0.0251	41,354 (71.60)	42,011 (72.73)	0.0254
Other race	2371 (3.81)	4440 (4.02)	0.0109	2227 (3.86)	1947 (3.37)	0.0260
Unknown race	4784 (7.69)	10,579 (9.58)	0.0675	4648 (8.05)	4555 (7.89)	0.0059
*Ethnicity*						
Hispanic or Latino	3635 (5.84)	6149 (5.57)	0.0117	3358 (5.81)	3061 (5.30)	0.0224
Not Hispanic or Latino	47,098 (75.69)	79,097 (71.65)	0.0919	43,209 (74.81)	42,757 (74.02)	0.0179
Unknown ethnicity	11,494 (18.47)	25,154 (22.78)	0.1068	11,194 (19.38)	11,943 (20.68)	0.0324
*Married*	13,373 (21.49)	27,633 (25.03)	0.0838	12,788 (22.14)	12,729 (22.04)	0.0025
HPV vaccination	873 (1.40)	1147 (1.04)	0.0331	756 (1.31)	667 (1.16)	0.0140
Tobacco use	3408 (5.48)	10,931 (9.90)	0.1159	3367 (5.83)	3127 (5.41)	0.0141
Cervical cancer screening^a^	3479 (5.59)	8582 (7.77)	0.0619	3373 (5.84)	3112 (5.39)	0.0155
Gynecological examination^a^	3548 (5.70)	8787 (7.96)	0.0896	3433 (5.94)	3397 (5.88)	0.0026
BMI^b^	30.2 ± 8.11	29.8 ± 8.05	0.0471	30.2 ± 8.11	29.7 ± 8.13	0.0619

PSM: propensity score matching; DMT: disease-modifying therapy; SD: standard deviation; SMD: standardized mean difference; HPV: human papilloma virus; BMI: body mass index.

a Values are any time pre-index.

b BMI (kg/m^2^) values at baseline available for 29.35% of the DMT cohort and 44.22% of non-DMT cohort before matching and 31.35% and 30.98% after matching.

The DMT group had significantly increased risks of all seven outcomes evaluated (Table 2): HPV positivity (RR: 1.52, 95% CI: 1.35–172), cervical dysplasia (RR: 1.40, 95% CI: 1.31–1.49), cervical cancer (RR: 1.23, 95% CI: 1.03–1.46), cervicitis (RR: 1.25, 95% CI: 1.10–1.41), HSV positivity (RR: 1.29, 95% CI: 1.14–1.45), vulvovaginitis (RR: 1.30, 95% CI: 1.23–1.37), and Bartholin cysts/abscesses (RR: 1.37, 95% CI: 1.07–1.76) compared to the no DMT group (Figure 1).

**Table 2.** Gynecologic complication cases and risks in women with multiple sclerosis on versus off disease-modifying therapy, after propensity score matching.

Outcome	On DMT *n* (%)	Off DMT *n* (%)	RR (95% CI)	*p*-value (corrected)
HPV+	643 (1.11)	423 (0.73)	1.52 (1.35–172)	**<0.001**
Cervical dysplasia	2266 (3.92)	1620 (2.80)	1.40 (1.31–1.49)	**<0.001**
Cervical cancer	275 (0.48)	224 (0.39)	1.23 (1.03–1.46)	**0.0223**
Cervicitis	541 (0.94)	434 (0.75)	1.25 (1.10–1.41)	**0.0008**
HSV+	593 (1.03)	461 (0.80)	1.29 (1.14–1.45)	**<0.001**
Vulvovaginitis	3135 (5.45)	2414 (4.20)	1.30 (1.23–1.37)	**<0.001**
Bartholin cyst/abscess	148 (0.26)	108 (0.19)	1.37 (1.07–1.76)	**0.0144**

DMT: disease-modifying therapy; RR: risk ratio; CI: confidence interval; HPV+: human papilloma virus positivity; HSV+: human simplex virus positivity.

Bolded *p*-values are significant at *p* < 0.05.

**Figure 1.**
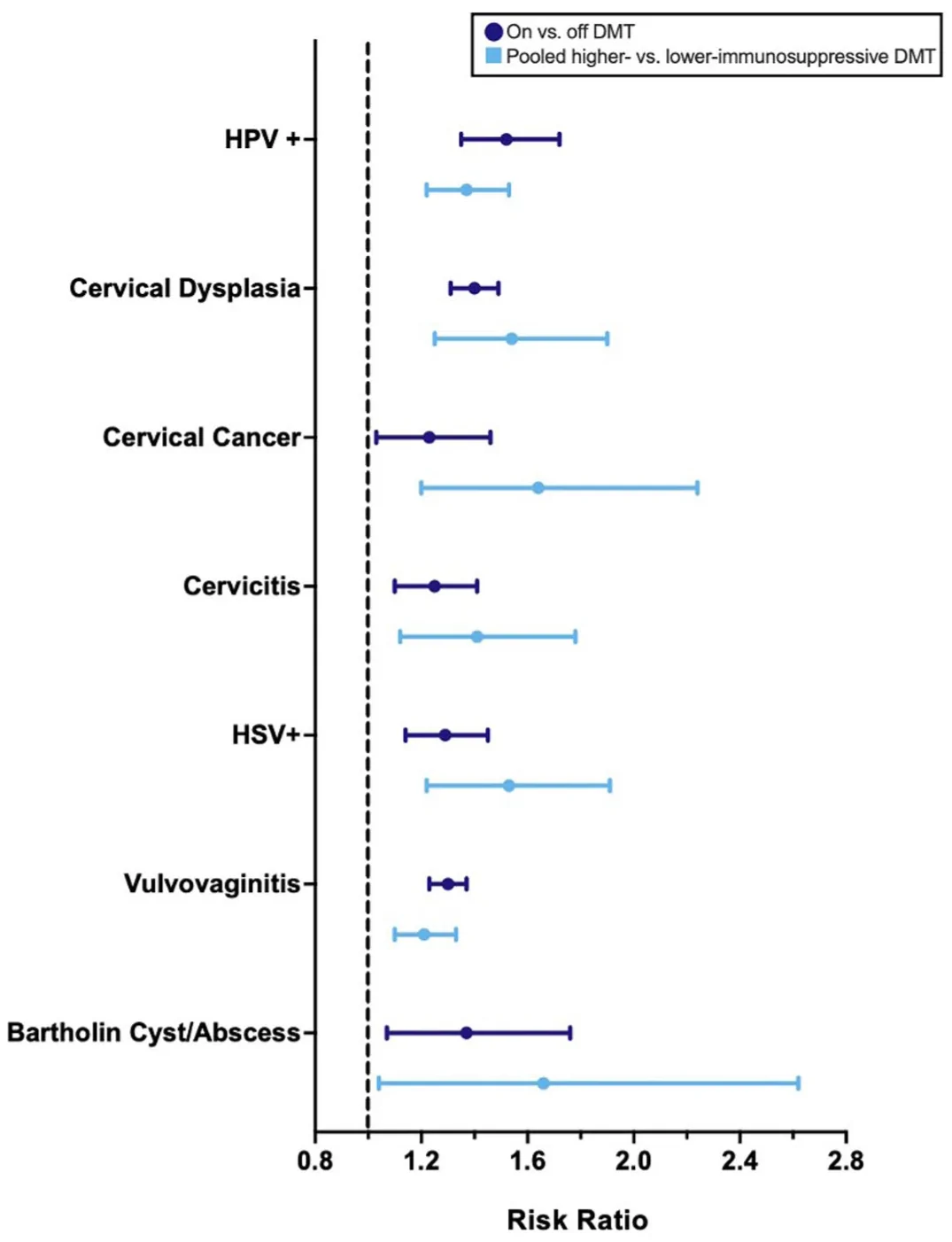
Forest plot illustrating risk ratios with 95% confidence intervals for gynecologic complications of multiple sclerosis disease-modifying therapies. Dashed line at one represents the reference point for statistical significance. HPV: human papilloma virus; HSV: herpes simplex virus; DMT: disease-modifying therapy. DMT classification: Higher-immunosuppressive (pooled high and moderate groups): anti-CD20 therapies, natalizumab, sphingosine-1-phosphate receptor modulators, fumarates, cladribine, alemtuzumab. Lower-immunosuppressive: glatiramer acetate, interferons, teriflunomide.

Our sensitivity analysis (*n* = 36,430 per group, cohorts with ⩾3 MS codes in a calendar year and healthcare engagement in the PSM) confirmed many of these findings, with increased risks observed in the DMT group for all outcomes except Bartholin cysts/abscesses and vulvovaginitis: HPV positivity (RR: 1.36, 95% CI: 1.04–1.79), cervical dysplasia (RR: 1.21, 95% CI: 1.12–1.31), cervical cancer (RR: 1.26, 95% CI: 1.02–1.46), cervicitis (RR: 1.28, 95% CI: 1.09–1.50), and HSV positivity (RR: 1.19, 95% CI: 1.03–1.36).

In a subanalysis comparing high- vs low-immunosuppressive therapies (*n* = 8231), high-immunosuppression was associated with greater risks of cervical dysplasia (RR: 1.29, 95% CI: 1.10–1.51), cervicitis (RR: 1.77, 95% CI: 1.25–2.49), HSV positivity (RR: 1.73, 95% CI: 1.25–2.40), and vulvovaginitis (RR: 1.27, 95% CI: 1.11–1.44). HPV positivity (RR: 34, 95% CI: 0.97–1.87) and Bartholin gland cysts/abscesses (RR: 1.73, 95% CI: 0.92–3.27) trended toward higher risk in the high-immunosuppression group, while cervical cancer risk was equivocal (Table 3).

**Table 3.** Subanalyses of gynecologic complication risk by level of immunosuppression.

Outcome	High vs low (*n* = 8,231)		Moderate vs low (*n* = 13,126)		High vs moderate (*n* = 9,737)		Moderate-to-high vs low (*n* = 16,328)	
	RR (95% CI)	*p*-value	RR (95% CI)	*p*-value	RR (95% CI)	*p*-value	RR (95% CI)	*p*-value
HPV+	1.34 (0.97–1.87)	0.1000	1.34 (1.05–1.71)	**0.0427**	1.09 (0.82–1.45)	0.6482	1.37 (1.22–1.53)	**0.0002**
Cervical dysplasia	1.29 (1.10–1.51)	**0.0033**	1.21 (1.07–1.38)	**0.0168**	1.10 (0.95–1.26)	0.3640	1.54 (1.25–1.90)	**0.0002**
Cervical cancer	1.29 (0.81–2.06)	0.2817	1.56 (1.09–2.22)	**0.0427**	0.96 (0.62–1.46)	0.8335	1.64 (1.20–2.24)	**0.0003**
Cervicitis	1.77 (1.25–2.49)	**0.0023**	1.20 (0.92–1.57)	0.2433	1.18 (0.88–1.59)	0.3640	1.41 (1.12–1.78)	**0.0044**
HSV+	1.73 (1.25–2.40)	**0.0023**	1.22 (0.94–1.58)	0.2433	1.45 (1.08–1.94)	**0.0480**	1.53 (1.22–1.91)	**0.0004**
Vulvovaginitis	1.27 (1.11–1.44)	**0.0021**	0.97 (0.87–1.08)	0.5745	1.50 (1.32–1.69)	**0.0007**	1.21 (1.10–1.33)	**0.0002**
Bartholin cyst/abscess	1.73 (0.92–3.27)	0.1000	1.26 (0.76–2.09)	0.4312	1.67 (0.93–2.99)	0.1934	1.66 (1.04–2.62)	**0.0302**

DMT classification: low (glatiramer acetate, interferons, teriflunomide), moderate (natalizumab, sphingosine-1-phosphate receptor modulators, fumarates, cladribine, alemtuzumab), and high immunosuppression (anti-CD20 therapies).

Each column represents an independent propensity score-matched analysis performed on the original unmatched cohorts for that specific pairwise comparison.

*p*-values are corrected and bolded if significant at *p* < 0.05.

When comparing moderate- vs low-immunosuppressive therapies (*n* = 13,126 per group), moderate immunosuppression was associated with higher risks of HPV positivity (RR: 1.34, 95% CI: 1.05–1.71), cervical dysplasia (RR: 1.21, 95% CI: 1.07–1.38), and cervical cancer (RR: 1.56, 95% CI: 1.09–2.22). Risks of HSV positivity, cervicitis, Bartholin gland cysts/abscesses, and vulvovaginitis were equivocal (Table 3).

When pooling moderate- and high-immunosuppression therapies and comparing with low-immunosuppression therapies (*n* = 16,328 per group), moderate/high immunosuppression was associated with higher risks of all seven outcomes (Table 3). Interestingly, only HSV positivity (RR: 1.45, 95% CI: 1.08–1.94) and vulvovaginitis (RR: 1.50, 95% CI: 1.32–1.69) risks were higher with high versus moderate immunosuppression (*n* = 9737 per group); all other outcome risks were not significantly different.

## Discussion

This multicenter, retrospective, propensity score-matched study of 115,522 women with MS demonstrated increased risks of several gynecologic complications with any use of DMTs. Specifically, DMT use was overall associated with higher risks of HPV positivity, cervical dysplasia, cervical cancer, cervicitis, HSV positivity, vulvovaginitis, and Bartholin cysts/abscesses. Altogether, this suggests that the chronic immunomodulation from MS DMTs may come with gynecologic sequelae, including by impairing HPV clearance and thus predisposing to cervical dysplasia and later malignancy.^3,5^

Furthermore, subanalyses stratified by degree of immunosuppression revealed that moderate- and high-immunosuppressive therapies (when pooled together) were associated with significantly greater risks of all outcomes compared with low-immunosuppressive therapies. However, the moderate- and high-immunosuppressive groups showed few differences from each other when independently compared with low-immunosuppressive therapies. Taken together, this pattern suggests that escalating beyond lower-immunosuppressive therapies (glatiramer, teriflunomide, and interferons) may be associated with greater risks of gynecologic pathology. Thus, future studies incorporating immunoprofiling of the female genital tract while on DMTs are warranted to further explain the mechanisms underlying our findings.^12^

Our study has several limitations. First, EHR-derived data are subject to misclassification and incomplete capture of care received outside contributing organizations, which could explain the large cohort of women with an MS diagnosis not on any DMT. However, such misclassification would bias our findings toward the null; prior research does report that women with MS are significantly less exposed to DMTs compared with men and people with MS in 72% of countries (some of which contribute to TriNetX) face barriers accessing DMTs.^27–29^ Nevertheless, selection bias, reporting bias (physicians may be more likely to document infectious outcomes in women receiving DMTs), patients dropped during PSM, and unmeasured confounders may still have influenced our findings. In addition, variables such as HPV vaccination, cervical cancer screening, gynecologic examinations, as well as outcome measures are likely underreported in EHR-derived data, which could explain the low observed rates. Furthermore, we could not evaluate DMT exposure duration, MS duration, treatment switching, or the DMT breakdown of our cohort as the TriNetX platform does not have reliable methods for doing so. Variation in testing practices/access across centers/regions may have also contributed to differential outcome detection. Finally, more advanced causal inference approaches, including doubly robust methods (not currently available in TriNetX), may offer advantages over PSM and should be considered in future studies, as should stratifying by race, ethnicity, tobacco use, and BMI.

In summary, this study of more than 100,000 women with MS adds to a growing body of work linking MS DMTs to gynecologic pathology.^5–12,30–34^ Although the absolute outcome risks we observed were low, their potential consequences, particularly cervical dysplasia and cancer, are clinically relevant. Thus, clinicians must integrate gynecological counseling and screening into MS management. To minimize HPV-related risks, DMT counseling can focus on safe sex, HPV vaccination (at least until age 45),^35^ and cancer screening per immunocompromised guidelines (more frequent screening to begin within 1 year of the onset of sexual activity, and throughout one’s whole life rather than stopping at age 65).^36^ While such screening can prevent progression to cancer, it is not without risk, including pain and anxiety.^37^ Therefore, prevention of HPV infection and reactivation should remain the primary goal when managing the gynecological risks of DMTs.

## Supplemental Material

sj-docx-1-msj-10.1177_13524585261475100 – Supplemental material for Gynecologic complications of disease-modifying therapy in women with multiple sclerosis Supplemental material, sj-docx-1-msj-10.1177_13524585261475100 for Gynecologic complications of disease-modifying therapy in women with multiple sclerosis by Alexandra Balshi, John P Dempsey, Yanli Zhang-James and Riley Bove in Multiple Sclerosis Journal

## Data Availability

De-identified data and statistical analyses may be shared with qualified investigators on reasonable request to the corresponding author.
