# Supplementary material for: Gynecologic complications of disease-modifying therapy in women with multiple sclerosis
Source: Mult Scler. 2026 Aug 13;32(12):1315–22. doi: 10.1177/13524585261475100 (PMC13672867; doi:10.1177/13524585261475100)
Supplement: Supplementary file 1 [file sj-docx-1-msj-10.1177_13524585261475100.docx]

**Supplementary Table 1. Coding used to define study variables.**

| **Diagnosis codes** |  |
| --- | --- |
| Multiple sclerosis | ICD-10: G35 |
| Human immunodeficiency virus disease | ICD-10: B20 |
| Transplanted organ and tissue status | ICD-10: Z94 |
| Family history of malignant neoplasm of other genital organs | ICD-10: Z80.49 |
| **DMTs** |  |
| Alemtuzumab | RXNORM:117055  HCPCS: J0202, J9010, Q9979 |
| Natalizumab | RXNORM:354770  HCPCS: J2323 |
| Ocrelizumab | RXNORM:1876366  HCPCS: J2350, C9494 |
| Ofatumumab | RXNORM:712566  HCPCS: J9302 |
| Rituximab | RXNORM:121191  HCPCS: J9310, J9312, Q5119, J9311, Q5123, C9467 |
| Ublitixumab | RXNORM: 2626349, OMOP5179597  HCPCS: J2329 |
| Fingolimod | RXNORM: 1012892 |
| Ozanimod | RXNORM: 2288236 |
| Siponimod | RXNORM: 2121085 |
| Ponesimod | RXNORM: 2532300 |
| Dimethyl fumarate | RXNORM: 1373478 |
| Diroximel fumarate | RXNORM: 2261783 |
| Cladribine | RXNORM: 44157  HCPCS: J9065 |
| Glatiramer acetate | RXNORM: 214582  HCPCS: J1595 |
| Teriflunomide | RXNORM: 1310520 |
| Interferon beta-1a | RXNORM: 75917 |
| Interferon beta-1b | RXNORM: 72257 |
| Peginterferon beta-1a | RXNORM: 1546168 |
| **Outcome codes** |  |
| HPV positivity | ICD-10: R87.82, B97.7  LOINC: 17399-7, 17401-1, 82354-2, 105077-2, 14499-8, 61373-7, 61372-9, 77399-4, 77400-0, 44550-2, 49891-5, 6516-9, 95536-9, 95539-3, 95538-5, 95537-7, 935535-1, 95534-4, 95533-6, 91852-4 |
| HSV positivity | ICD-10: A60  LOINC: 5851-1 |
| Cervical dysplasia | ICD-10: R87.6, N87 |
| Cervical cancer | ICD-10: C53, D06, D26, R87.614 |
| Cervicitis | ICD-10: N72  LOINC: 14475-8, 12281-2 |
| Bartholin cyst/abscess | ICD-10: N75.0, N75.1 |
| Vulvovaginitis | ICD-10: N76 |
| **PSM criteria** |  |
| Tobacco use | ICD-10: Z72.0, F17 |
| Socioeconomic status | ICD-10: Z55-Z65 |
| Healthcare engagement | ICD-10: Z00-Z13 |
| HPV vaccination | CVX: 137 |
| Cervical cancer screening | CPT: 88142 |
| Hemoglobin A1c | LOINC: 4548-4, 17855-8, 4549-2, 17856-6 |
| Corticosteroid use | ATC: H02 |
| Antibiotic use | ATC: J01 |

Abbreviations: ICD-10, International Classification of Diseases, Tenth Revision; DMT, disease-modifying therapy; HCPCS, Healthcare Common Procedure Coding System; HPV, human papilloma virus; PSM, propensity score matching; CPT, Current Procedural Terminology; ATC, Anatomical Therapeutic Chemical; CVX, Vaccines Administered.

**Supplementary Table 2. Baseline characteristics of sensitivity analysis comparing cohorts of women with multiple sclerosis on versus off disease-modifying therapy, before and after propensity score matching.**

| **Cohort 1 and cohort 2 patient count before and after propensity score matching** | | | | | | | | | | | | | |
| --- | --- | --- | --- | --- | --- | --- | --- | --- | --- | --- | --- | --- | --- |
|  | | | Cohort | | | Patient count before matching | | | | | Patient count after matching | | |
|  | | | 1 – On DMT | | | 57,828 | | | | | 36,430 | | |
|  | | | 2 – Off DMT | | | 37,889 | | | | | 36,430 | | |
| **Cohort 1 (N = 57,828) and cohort 2 (N = 37,889) characteristics before propensity score matching** | | | | | | | | | | | | | |
|  | **Demographics** | | | | | | | | | | | | |
|  |  | Cohort | | |  | Mean ± SD | Median | IQR | Patients | % of Cohort | | P-Value | Std diff. |
|  |  | 1 2 | | Age | Current Age | 52.9 +/- 12.7 55.5 +/- 13.0 | 54 57 | 20 20 | 57,828 37,889 | 100% 100% | | <0.001 | 0.202 |
|  |  | 1 2 | | AI | Age at Index | 43.5 +/- 11.5 47.2 +/- 11.7 | 44 49 | 19 19 | 57,828 37,889 | 100% 100% | | <0.001 | 0.323 |
|  |  | 1 2 | | 2054-5 | Black or African American |  |  |  | 9,570 6,330 | 16.5% 16.7% | | 0.522 | 0.004 |
|  |  | 1 2 | | M | Married |  |  |  | 16,126 11,321 | 27.9% 29.9% | | <0.001 | 0.044 |
|  |  | 1 2 | | 2106-3 | White |  |  |  | 41,683 26,527 | 72.1% 70.0% | | <0.001 | 0.046 |
|  |  | 1 2 | | 1002-5 | Native American or Alaska Native |  |  |  | 262 183 | 0.5% 0.5% | | 0.506 | 0.004 |
|  |  | 1 2 | | UNK | Unknown Race |  |  |  | 3,944 3,028 | 6.8% 8.0% | | <0.001 | 0.045 |
|  |  | 1 2 | | S | Never Married |  |  |  | 9,152 7,122 | 15.8% 18.8% | | <0.001 | 0.079 |
|  |  | 1 2 | | 2076-8 | Native Hawaiian or Other Pacific Islander |  |  |  | 59 55 | 0.1% 0.1% | | 0.058 | 0.012 |
|  |  | 1 2 | | UN | Unknown Ethnicity |  |  |  | 13,780 10,049 | 23.8% 26.5% | | <0.001 | 0.062 |
|  |  | 1 2 | | 2186-5 | Not Hispanic or Latino |  |  |  | 40,807 26,212 | 70.6% 69.2% | | <0.001 | 0.030 |
|  |  | 1 2 | | 2135-2 | Hispanic or Latino |  |  |  | 3,241 1,628 | 5.6% 4.3% | | <0.001 | 0.060 |
|  |  | 1 2 | | 2131-1 | Other Race |  |  |  | 1,774 1,282 | 3.1% 3.4% | | 0.007 | 0.018 |
|  |  | 1 2 | | 2028-9 | Asian |  |  |  | 536 484 | 0.9% 1.3% | | <0.001 | 0.034 |
|  | **Diagnosis** | | | | | | | | | | | | |
|  |  | Cohort | | |  | Mean ± SD | Median | IQR | Patients | % of Cohort | | P-Value | Std diff. |
|  |  | 1 2 | | Z72.0 | Tobacco use |  |  |  | 612 661 | 1.1% 1.7% | | <0.001 | 0.058 |
|  |  | 1 2 | | F17 | Nicotine dependence |  |  |  | 2,623 2,411 | 4.5% 6.4% | | <0.001 | 0.081 |
|  |  | 1 2 | | Z01.4 | Encounter for gynecological examination |  |  |  | 3,708 2,581 | 6.4% 6.8% | | 0.015 | 0.016 |
|  |  | 1 2 | | Z12.4 | Encounter for screening for malignant neoplasm of cervix |  |  |  | 2,845 1,893 | 4.9% 5.0% | | 0.594 | 0.004 |
|  |  | 1 2 | | Z55-Z65 | Persons with potential health hazards related to socioeconomic and psychosocial circumstances |  |  |  | 495 397 | 0.9% 1.0% | | 0.003 | 0.020 |
|  |  | 1 2 | | Z00-Z13 | Persons encountering health services for examinations |  |  |  | 11,832 9,191 | 20.5% 24.3% | | <0.001 | 0.091 |
|  | **Medication** | | | | | | | | | | | | |
|  |  | Cohort | | |  | Mean ± SD | Median | IQR | Patients | % of Cohort | | P-Value | Std diff. |
|  |  | 1 2 | | 137 | HPV |  |  |  | 913 334 | 1.6% 0.9% | | <0.001 | 0.063 |
|  | **Laboratory** | | | | | | | | | | | | |
|  |  | Cohort | | |  | Mean ± SD | Median | IQR | Patients | % of Cohort | | P-Value | Std diff. |
|  |  | 1 2 | | 9083 | BMI | 30.2 +/- 8.1 30.1 +/- 8.1 | 28.799 28.697 | 11 10.720 | 20,770 14,039 | 35.9% 37.1% | | 0.408 | 0.009 |
| **Cohort 1 (N = 36,430) and cohort 2 (N = 36,430) characteristics after propensity score matching** | | | | | | | | | | | | | |
|  | **Demographics** | | | | | | | | | | | | |
|  |  | Cohort | | |  | Mean ± SD | Median | IQR | Patients | % of Cohort | | P-Value | Std diff. |
|  |  | 1 2 | | Age | Current Age | 56.1 +/- 12.5 55.1 +/- 13.0 | 58 56 | 19 21 | 36,430 36,430 | 100% 100% | | <0.001 | 0.079 |
|  |  | 1 2 | | AI | Age at Index | 46.6 +/- 11.4 46.7 +/- 11.7 | 48 48 | 18 19 | 36,430 36,430 | 100% 100% | | 0.186 | 0.010 |
|  |  | 1 2 | | 2054-5 | Black or African American |  |  |  | 6,331 6,075 | 17.4% 16.7% | | 0.012 | 0.019 |
|  |  | 1 2 | | M | Married |  |  |  | 10,956 10,845 | 30.1% 29.8% | | 0.369 | 0.007 |
|  |  | 1 2 | | 2106-3 | White |  |  |  | 25,406 25,720 | 69.7% 70.6% | | 0.011 | 0.019 |
|  |  | 1 2 | | 1002-5 | Native American or Alaska Native |  |  |  | 164 167 | 0.5% 0.5% | | 0.869 | 0.001 |
|  |  | 1 2 | | UNK | Unknown Race |  |  |  | 2,904 2,774 | 8.0% 7.6% | | 0.072 | 0.013 |
|  |  | 1 2 | | S | Never Married |  |  |  | 6,681 6,544 | 18.3% 18.0% | | 0.188 | 0.010 |
|  |  | 1 2 | | 2076-8 | Native Hawaiian or Other Pacific Islander |  |  |  | 35 51 | 0.1% 0.1% | | 0.084 | 0.013 |
|  |  | 1 2 | | UN | Unknown Ethnicity |  |  |  | 9,989 9,509 | 27.4% 26.1% | | <0.001 | 0.030 |
|  |  | 1 2 | | 2186-5 | Not Hispanic or Latino |  |  |  | 24,878 25,315 | 68.3% 69.5% | | <0.001 | 0.026 |
|  |  | 1 2 | | 2135-2 | Hispanic or Latino |  |  |  | 1,563 1,606 | 4.3% 4.4% | | 0.435 | 0.006 |
|  |  | 1 2 | | 2131-1 | Other Race |  |  |  | 1,153 1,215 | 3.2% 3.3% | | 0.195 | 0.010 |
|  |  | 1 2 | | 2028-9 | Asian |  |  |  | 437 428 | 1.2% 1.2% | | 0.758 | 0.002 |
|  | **Diagnosis** | | | | | | | | | | | | |
|  |  | Cohort | | |  | Mean ± SD | Median | IQR | Patients | % of Cohort | | P-Value | Std diff. |
|  |  | 1 2 | | Z72.0 | Tobacco use |  |  |  | 505 529 | 1.4% 1.5% | | 0.452 | 0.006 |
|  |  | 1 2 | | F17 | Nicotine dependence |  |  |  | 1,978 2,093 | 5.4% 5.7% | | 0.064 | 0.014 |
|  |  | 1 2 | | Z01.4 | Encounter for gynecological examination |  |  |  | 2,416 2,440 | 6.6% 6.7% | | 0.721 | 0.003 |
|  |  | 1 2 | | Z12.4 | Encounter for screening for malignant neoplasm of cervix |  |  |  | 1,782 1,777 | 4.9% 4.9% | | 0.932 | 0.001 |
|  |  | 1 2 | | Z55-Z65 | Persons with potential health hazards related to socioeconomic and psychosocial circumstances |  |  |  | 306 356 | 0.8% 1.0% | | 0.051 | 0.014 |
|  |  | 1 2 | | Z00-Z13 | Persons encountering health services for examinations |  |  |  | 7,732 8,651 | 21.2% 23.7% | | <0.001 | 0.060 |
|  | **Medication** | | | | | | | | | | | | |
|  |  | Cohort | | |  | Mean ± SD | Median | IQR | Patients | % of Cohort | | P-Value | Std diff. |
|  |  | 1 2 | | 137 | HPV |  |  |  | 289 334 | 0.8% 0.9% | | 0.070 | 0.013 |
|  | **Laboratory** | | | | | | | | | | | | |
|  |  | Cohort | | |  | Mean ± SD | Median | IQR | Patients | % of Cohort | | P-Value | Std diff. |
|  |  | 1 2 | | 9083 | BMI | 30.2 +/- 8.0 30.2 +/- 8.2 | 28.955 28.778 | 10.819 10.897 | 13,289 13,294 | 36.5% 36.5% | | 0.520 | 0.008 |

Abbreviations: DMT, disease-modifying therapy; SD, standard deviation; std. diff,, standardized mean difference; HPV, human papilloma virus; BMI, body mass index; IQR, interquartile range.

**Supplementary Table 3. Disease-modifying therapy categorization rationale.**

| **Immunosuppression Category** | **DMT Class** | **Agents** | **Rationale** |
| --- | --- | --- | --- |
| **Low** | Interferons | Interferon beta-1a, interferon beta-1b, peginterferon beta-1a | Shifts immune profile without lymphodepletion/immunosuppression.^1^ |
|  | Glatiramer acetate | Glatiramer acetate | Shifts immune profile without lymphodepletion/immunosuppression.^1^ |
|  | Pyrimidine synthesis inhibitor | Teriflunomide | Limits T- and B-cell proliferation; minimal lymphodepletion/immunosuppression.^2^ |
| **Moderate** | S1P receptor modulators | Fingolimod, siponimod, ozanimod, ponesimod | Sequesters lymphocytes in lymph nodes, reducing circulating counts; moderate infection risk.^1^ |
|  | Fumarates | Dimethyl fumarate, diroximel fumarate, monomethyl fumarate | Moderate lymphodepletion (especially CD8+), moderate infection risk.^3^ |
|  | α4-integrin inhibitor | Natalizumab | Blocks immune cell trafficking into the CNS without systemic depletion; associated with opportunistic infections.^1^ |
|  | Immune reconstitution therapies | Cladribine, alemtuzumab | Induces transient though profound lymphodepletion followed by immune reconstitution; effects are time-limited.^4^ |
| **High** | Anti-CD20 monoclonal antibodies | Ocrelizumab, ofatumumab, rituximab, ublituximab | Sustained B-cell depletion; higher infection risk.^1^ |
| Abbreviations: DMT, disease-modifying therapy; S1P, sphingosine-1-phosphate; PML, progressive multifocal leukoencephalopathy.   1. Luna G, Alping P, Burman J, et al. Infection Risks Among Patients With Multiple Sclerosis Treated With Fingolimod, Natalizumab, Rituximab, and Injectable Therapies. *JAMA Neurol*. 2020;77(2):184-191. doi:10.1001/jamaneurol.2019.3365 2. Bar-Or A, Pachner A, Menguy-Vacheron F, Kaplan J, Wiendl H. Teriflunomide and its mechanism of action in multiple sclerosis. *Drugs*. 2014;74(6):659-674. doi:10.1007/s40265-014-0212-x 3. Spencer CM, Crabtree-Hartman EC, Lehmann-Horn K, Cree BA, Zamvil SS. Reduction of CD8(+) T lymphocytes in multiple sclerosis patients treated with dimethyl fumarate. *Neurol Neuroimmunol Neuroinflamm*. 2015;2(3):e76. Published 2015 Feb 12. doi:10.1212/NXI.0000000000000076 4. Baker D, Herrod SS, Alvarez-Gonzalez C, Zalewski L, Albor C, Schmierer K. Both cladribine and alemtuzumab may effect MS via B-cell depletion. *Neurol Neuroimmunol Neuroinflamm*. 2017;4(4):e360. Published 2017 Jun 5. doi:10.1212/NXI.0000000000000360 | | | |

**Supplementary Table 4. Baseline characteristics of cohorts of women with multiple sclerosis on high- versus low-immunosuppression, before and after propensity score matching.**

| **Cohort 1 and cohort 2 patient count before and after propensity score matching** | | | | | | | | | | | | | |
| --- | --- | --- | --- | --- | --- | --- | --- | --- | --- | --- | --- | --- | --- |
|  | | | Cohort | | | Patient count before matching | | | | | Patient count after matching | | |
|  | | | 1 – High-immunosuppression | | | 14,917 | | | | | 8,231 | | |
|  | | | 2 – Low-immunosuppression | | | 18,963 | | | | | 8,231 | | |
| **Cohort 1 (N = 14,917) and cohort 2 (N = 18,963) characteristics before propensity score matching** | | | | | | | | | | | | | |
|  | **Demographics** | | | | | | | | | | | | |
|  |  | Cohort | | |  | Mean ± SD | Median | IQR | Patients | % of Cohort | | P-Value | Std diff. |
|  |  | 1 2 | | Age | Current Age | 46.9 +/- 12.3 57.8 +/- 11.5 | 46 59 | 19 17 | 14,917 18,963 | 100% 100% | | <0.001 | 0.922 |
|  |  | 1 2 | | AI | Age at Index | 41.2 +/- 11.2 47.5 +/- 10.9 | 41 49 | 18 16 | 14,917 18,963 | 100% 100% | | <0.001 | 0.572 |
|  |  | 1 2 | | 2054-5 | Black or African American |  |  |  | 2,575 2,558 | 17.3% 13.5% | | <0.001 | 0.105 |
|  |  | 1 2 | | M | Married |  |  |  | 2,985 4,292 | 20.0% 22.6% | | <0.001 | 0.064 |
|  |  | 1 2 | | 2106-3 | White |  |  |  | 10,368 14,172 | 69.5% 74.7% | | <0.001 | 0.117 |
|  |  | 1 2 | | 1002-5 | Native American or Alaska Native |  |  |  | 72 62 | 0.5% 0.3% | | 0.023 | 0.025 |
|  |  | 1 2 | | UNK | Unknown Race |  |  |  | 947 1,416 | 6.3% 7.5% | | <0.001 | 0.044 |
|  |  | 1 2 | | S | Never Married |  |  |  | 1,910 1,851 | 12.8% 9.8% | | <0.001 | 0.096 |
|  |  | 1 2 | | 2076-8 | Native Hawaiian or Other Pacific Islander |  |  |  | 17 13 | 0.1% 0.1% | | 0.163 | 0.015 |
|  |  | 1 2 | | UN | Unknown Ethnicity |  |  |  | 2,377 3,997 | 15.9% 21.1% | | <0.001 | 0.133 |
|  |  | 1 2 | | 2186-5 | Not Hispanic or Latino |  |  |  | 11,356 14,134 | 76.1% 74.5% | | 0.001 | 0.037 |
|  |  | 1 2 | | 2135-2 | Hispanic or Latino |  |  |  | 1,184 832 | 7.9% 4.4% | | <0.001 | 0.148 |
|  |  | 1 2 | | 2131-1 | Other Race |  |  |  | 758 616 | 5.1% 3.2% | | <0.001 | 0.092 |
|  |  | 1 2 | | 2028-9 | Asian |  |  |  | 180 126 | 1.2% 0.7% | | <0.001 | 0.056 |
|  | **Diagnosis** | | | | | | | | | | | | |
|  |  | Cohort | | |  | Mean ± SD | Median | IQR | Patients | % of Cohort | | P-Value | Std diff. |
|  |  | 1 2 | | Z72.0 | Tobacco use |  |  |  | 216 115 | 1.4% 0.6% | | <0.001 | 0.084 |
|  |  | 1 2 | | F17 | Nicotine dependence |  |  |  | 920 714 | 6.2% 3.8% | | <0.001 | 0.111 |
|  |  | 1 2 | | Z01.4 | Encounter for gynecological examination |  |  |  | 1,101 939 | 7.4% 5.0% | | <0.001 | 0.101 |
|  |  | 1 2 | | Z12.4 | Encounter for screening for malignant neoplasm of cervix |  |  |  | 879 702 | 5.9% 3.7% | | <0.001 | 0.103 |
|  |  | 1 2 | | Z55-Z65 | Persons with potential health hazards related to socioeconomic and psychosocial circumstances |  |  |  | 207 80 | 1.4% 0.4% | | <0.001 | 0.102 |
|  | **Medication** | | | | | | | | | | | | |
|  |  | Cohort | | |  | Mean ± SD | Median | IQR | Patients | % of Cohort | | P-Value | Std diff. |
|  |  | 1 2 | | 137 | HPV |  |  |  | 430 59 | 2.9% 0.3% | | <0.001 | 0.206 |
|  | **Laboratory** | | | | | | | | | | | | |
|  |  | Cohort | | |  | Mean ± SD | Median | IQR | Patients | % of Cohort | | P-Value | Std diff. |
|  |  | 1 2 | | 9083 | BMI | 30.1 +/- 8.2 30.0 +/- 7.9 | 28.579 28.622 | 11.028 10.410 | 6,291 4,657 | 42.2% 24.6% | | 0.700 | 0.007 |
| **Cohort 1 (N = 8,231) and cohort 2 (N = 8,231) characteristics after propensity score matching** | | | | | | | | | | | | | |
|  | **Demographics** | | | | | | | | | | | | |
|  |  | Cohort | | |  | Mean ± SD | Median | IQR | Patients | % of Cohort | | P-Value | Std diff. |
|  |  | 1 2 | | Age | Current Age | 52.5 +/- 11.1 52.6 +/- 11.4 | 53 53 | 17 18 | 8,231 8,231 | 100% 100% | | 0.876 | 0.002 |
|  |  | 1 2 | | AI | Age at Index | 44.8 +/- 10.8 44.8 +/- 11.4 | 45 45 | 17 18 | 8,231 8,231 | 100% 100% | | 0.994 | <0.001 |
|  |  | 1 2 | | 2054-5 | Black or African American |  |  |  | 1,346 1,307 | 16.4% 15.9% | | 0.408 | 0.013 |
|  |  | 1 2 | | M | Married |  |  |  | 1,856 1,862 | 22.5% 22.6% | | 0.911 | 0.002 |
|  |  | 1 2 | | 2106-3 | White |  |  |  | 5,887 5,964 | 71.5% 72.5% | | 0.181 | 0.021 |
|  |  | 1 2 | | 1002-5 | Native American or Alaska Native |  |  |  | 24 28 | 0.3% 0.3% | | 0.578 | 0.009 |
|  |  | 1 2 | | UNK | Unknown Race |  |  |  | 537 509 | 6.5% 6.2% | | 0.371 | 0.014 |
|  |  | 1 2 | | S | Never Married |  |  |  | 974 969 | 11.8% 11.8% | | 0.904 | 0.002 |
|  |  | 1 2 | | 2076-8 | Native Hawaiian or Other Pacific Islander |  |  |  | 10 10 | 0.1% 0.1% | | 1 | <0.001 |
|  |  | 1 2 | | UN | Unknown Ethnicity |  |  |  | 1,489 1,487 | 18.1% 18.1% | | 0.968 | 0.001 |
|  |  | 1 2 | | 2186-5 | Not Hispanic or Latino |  |  |  | 6,278 6,288 | 76.3% 76.4% | | 0.855 | 0.003 |
|  |  | 1 2 | | 2135-2 | Hispanic or Latino |  |  |  | 464 456 | 5.6% 5.5% | | 0.786 | 0.004 |
|  |  | 1 2 | | 2131-1 | Other Race |  |  |  | 357 341 | 4.3% 4.1% | | 0.536 | 0.010 |
|  |  | 1 2 | | 2028-9 | Asian |  |  |  | 71 75 | 0.9% 0.9% | | 0.739 | 0.005 |
|  | **Diagnosis** | | | | | | | | | | | | |
|  |  | Cohort | | |  | Mean ± SD | Median | IQR | Patients | % of Cohort | | P-Value | Std diff. |
|  |  | 1 2 | | Z72.0 | Tobacco use |  |  |  | 99 93 | 1.2% 1.1% | | 0.663 | 0.007 |
|  |  | 1 2 | | F17 | Nicotine dependence |  |  |  | 442 403 | 5.4% 4.9% | | 0.168 | 0.021 |
|  |  | 1 2 | | Z01.4 | Encounter for gynecological examination |  |  |  | 492 528 | 6.0% 6.4% | | 0.244 | 0.018 |
|  |  | 1 2 | | Z12.4 | Encounter for screening for malignant neoplasm of cervix |  |  |  | 358 393 | 4.3% 4.8% | | 0.191 | 0.020 |
|  |  | 1 2 | | Z55-Z65 | Persons with potential health hazards related to socioeconomic and psychosocial circumstances |  |  |  | 66 58 | 0.8% 0.7% | | 0.471 | 0.011 |
|  | **Medication** | | | | | | | | | | | | |
|  |  | Cohort | | |  | Mean ± SD | Median | IQR | Patients | % of Cohort | | P-Value | Std diff. |
|  |  | 1 2 | | 137 | HPV |  |  |  | 50 58 | 0.6% 0.7% | | 0.440 | 0.012 |
|  | **Laboratory** | | | | | | | | | | | | |
|  |  | Cohort | | |  | Mean ± SD | Median | IQR | Patients | % of Cohort | | P-Value | Std diff. |
|  |  | 1 2 | | 9083 | BMI | 29.5 +/- 8.0 30.4 +/- 8.1 | 27.990 29 | 10.571 10.873 | 2,867 2,949 | 34.8% 35.8% | | <0.001 | 0.120 |

Abbreviations: DMT, disease-modifying therapy; SD, standard deviation; std. diff,, standardized mean difference; HPV, human papilloma virus; BMI, body mass index; IQR, interquartile range.

**Supplementary Table 5. Baseline characteristics of cohorts of women with multiple sclerosis on moderate- versus low-immunosuppression, before and after propensity score matching.**

| **Cohort 1 and cohort 2 patient count before and after propensity score matching** | | | | | | | | | | | | | |
| --- | --- | --- | --- | --- | --- | --- | --- | --- | --- | --- | --- | --- | --- |
|  | | | Cohort | | | Patient count before matching | | | | | Patient count after matching | | |
|  | | | 1 – Moderate-immunosuppression | | | 15,521 | | | | | 13,126 | | |
|  | | | 2 – Low-immunosuppression | | | 18,963 | | | | | 13,126 | | |
| **Cohort 1 (N = 15,521) and cohort 2 (N = 18,963) characteristics before propensity score matching** | | | | | | | | | | | | | |
|  | **Demographics** | | | | | | | | | | | | |
|  |  | Cohort | | |  | Mean ± SD | Median | IQR | Patients | % of Cohort | | P-Value | Std diff. |
|  |  | 1 2 | | Age | Current Age | 51.8 +/- 11.8 57.8 +/- 11.5 | 52 59 | 18 17 | 15,521 18,963 | 100% 100% | | <0.001 | 0.519 |
|  |  | 1 2 | | AI | Age at Index | 42.9 +/- 11.1 47.5 +/- 10.9 | 43 49 | 17 16 | 15,521 18,963 | 100% 100% | | <0.001 | 0.419 |
|  |  | 1 2 | | 2054-5 | Black or African American |  |  |  | 2,353 2,558 | 15.2% 13.5% | | <0.001 | 0.048 |
|  |  | 1 2 | | M | Married |  |  |  | 3,623 4,292 | 23.3% 22.6% | | 0.119 | 0.017 |
|  |  | 1 2 | | 2106-3 | White |  |  |  | 10,824 14,172 | 69.7% 74.7% | | <0.001 | 0.112 |
|  |  | 1 2 | | 1002-5 | Native American or Alaska Native |  |  |  | 56 62 | 0.4% 0.3% | | 0.592 | 0.006 |
|  |  | 1 2 | | UNK | Unknown Race |  |  |  | 1,597 1,416 | 10.3% 7.5% | | <0.001 | 0.099 |
|  |  | 1 2 | | S | Never Married |  |  |  | 2,094 1,851 | 13.5% 9.8% | | <0.001 | 0.117 |
|  |  | 1 2 | | 2076-8 | Native Hawaiian or Other Pacific Islander |  |  |  | 18 13 | 0.1% 0.1% | | 0.144 | 0.016 |
|  |  | 1 2 | | UN | Unknown Ethnicity |  |  |  | 3,075 3,997 | 19.8% 21.1% | | 0.004 | 0.031 |
|  |  | 1 2 | | 2186-5 | Not Hispanic or Latino |  |  |  | 11,594 14,134 | 74.7% 74.5% | | 0.727 | 0.004 |
|  |  | 1 2 | | 2135-2 | Hispanic or Latino |  |  |  | 852 832 | 5.5% 4.4% | | <0.001 | 0.051 |
|  |  | 1 2 | | 2131-1 | Other Race |  |  |  | 528 616 | 3.4% 3.2% | | 0.429 | 0.009 |
|  |  | 1 2 | | 2028-9 | Asian |  |  |  | 145 126 | 0.9% 0.7% | | 0.005 | 0.030 |
|  | **Diagnosis** | | | | | | | | | | | | |
|  |  | Cohort | | |  | Mean ± SD | Median | IQR | Patients | % of Cohort | | P-Value | Std diff. |
|  |  | 1 2 | | Z72.0 | Tobacco use |  |  |  | 134 115 | 0.9% 0.6% | | 0.005 | 0.030 |
|  |  | 1 2 | | F17 | Nicotine dependence |  |  |  | 642 714 | 4.1% 3.8% | | 0.078 | 0.019 |
|  |  | 1 2 | | Z01.4 | Encounter for gynecological examination |  |  |  | 852 939 | 5.5% 5.0% | | 0.025 | 0.024 |
|  |  | 1 2 | | Z12.4 | Encounter for screening for malignant neoplasm of cervix |  |  |  | 620 702 | 4.0% 3.7% | | 0.159 | 0.015 |
|  |  | 1 2 | | Z55-Z65 | Persons with potential health hazards related to socioeconomic and psychosocial circumstances |  |  |  | 105 80 | 0.7% 0.4% | | 0.001 | 0.034 |
|  | **Medication** | | | | | | | | | | | | |
|  |  | Cohort | | |  | Mean ± SD | Median | IQR | Patients | % of Cohort | | P-Value | Std diff. |
|  |  | 1 2 | | 137 | HPV |  |  |  | 162 59 | 1.0% 0.3% | | <0.001 | 0.089 |
|  | **Laboratory** | | | | | | | | | | | | |
|  |  | Cohort | | |  | Mean ± SD | Median | IQR | Patients | % of Cohort | | P-Value | Std diff. |
|  |  | 1 2 | | 9083 | BMI | 30.0 +/- 8.1 30.0 +/- 7.9 | 28.662 28.622 | 11.019 10.410 | 4,578 4,657 | 29.5% 24.6% | | 0.901 | 0.003 |
| **Cohort 1 (N = 13,126) and cohort 2 (N = 13,126) characteristics after propensity score matching** | | | | | | | | | | | | | |
|  | **Demographics** | | | | | | | | | | | | |
|  |  | Cohort | | |  | Mean ± SD | Median | IQR | Patients | % of Cohort | | P-Value | Std diff. |
|  |  | 1 2 | | Age | Current Age | 54.1 +/- 10.9 54.1 +/- 11.1 | 55 55 | 16 17 | 13,126 13,126 | 100% 100% | | 0.884 | 0.002 |
|  |  | 1 2 | | AI | Age at Index | 44.7 +/- 10.6 44.6 +/- 10.9 | 45 45 | 16 17 | 13,126 13,126 | 100% 100% | | 0.794 | 0.003 |
|  |  | 1 2 | | 2054-5 | Black or African American |  |  |  | 1,896 1,909 | 14.4% 14.5% | | 0.820 | 0.003 |
|  |  | 1 2 | | M | Married |  |  |  | 3,122 3,151 | 23.8% 24.0% | | 0.675 | 0.005 |
|  |  | 1 2 | | 2106-3 | White |  |  |  | 9,459 9,516 | 72.1% 72.5% | | 0.432 | 0.010 |
|  |  | 1 2 | | 1002-5 | Native American or Alaska Native |  |  |  | 45 41 | 0.3% 0.3% | | 0.666 | 0.005 |
|  |  | 1 2 | | UNK | Unknown Race |  |  |  | 1,174 1,117 | 8.9% 8.5% | | 0.213 | 0.015 |
|  |  | 1 2 | | S | Never Married |  |  |  | 1,518 1,497 | 11.6% 11.4% | | 0.684 | 0.005 |
|  |  | 1 2 | | 2076-8 | Native Hawaiian or Other Pacific Islander |  |  |  | 12 12 | 0.1% 0.1% | | 1 | <0.001 |
|  |  | 1 2 | | UN | Unknown Ethnicity |  |  |  | 2,611 2,627 | 19.9% 20.0% | | 0.805 | 0.003 |
|  |  | 1 2 | | 2186-5 | Not Hispanic or Latino |  |  |  | 9,849 9,853 | 75.0% 75.1% | | 0.955 | 0.001 |
|  |  | 1 2 | | 2135-2 | Hispanic or Latino |  |  |  | 666 646 | 5.1% 4.9% | | 0.571 | 0.007 |
|  |  | 1 2 | | 2131-1 | Other Race |  |  |  | 438 430 | 3.3% 3.3% | | 0.782 | 0.003 |
|  |  | 1 2 | | 2028-9 | Asian |  |  |  | 102 101 | 0.8% 0.8% | | 0.944 | 0.001 |
|  | **Diagnosis** | | | | | | | | | | | | |
|  |  | Cohort | | |  | Mean ± SD | Median | IQR | Patients | % of Cohort | | P-Value | Std diff. |
|  |  | 1 2 | | Z72.0 | Tobacco use |  |  |  | 106 105 | 0.8% 0.8% | | 0.945 | 0.001 |
|  |  | 1 2 | | F17 | Nicotine dependence |  |  |  | 523 501 | 4.0% 3.8% | | 0.483 | 0.009 |
|  |  | 1 2 | | Z01.4 | Encounter for gynecological examination |  |  |  | 676 700 | 5.2% 5.3% | | 0.506 | 0.008 |
|  |  | 1 2 | | Z12.4 | Encounter for screening for malignant neoplasm of cervix |  |  |  | 483 517 | 3.7% 3.9% | | 0.273 | 0.014 |
|  |  | 1 2 | | Z55-Z65 | Persons with potential health hazards related to socioeconomic and psychosocial circumstances |  |  |  | 73 69 | 0.6% 0.5% | | 0.736 | 0.004 |
|  | **Medication** | | | | | | | | | | | | |
|  |  | Cohort | | |  | Mean ± SD | Median | IQR | Patients | % of Cohort | | P-Value | Std diff. |
|  |  | 1 2 | | 137 | HPV |  |  |  | 55 59 | 0.4% 0.4% | | 0.707 | 0.005 |
|  | **Laboratory** | | | | | | | | | | | | |
|  |  | Cohort | | |  | Mean ± SD | Median | IQR | Patients | % of Cohort | | P-Value | Std diff. |
|  |  | 1 2 | | 9083 | BMI | 29.9 +/- 8.0 30.2 +/- 8.0 | 28.513 28.935 | 10.910 10.771 | 3,584 3,692 | 27.3% 28.1% | | 0.045 | 0.047 |

Abbreviations: DMT, disease-modifying therapy; SD, standard deviation; std. diff,, standardized mean difference; HPV, human papilloma virus; BMI, body mass index; IQR, interquartile range.

**Supplementary Table 6. Baseline characteristics of cohorts of women with multiple sclerosis on pooled moderate- and high- versus low-immunosuppression, before and after propensity score matching.**

| **Cohort 1 and cohort 2 patient count before and after propensity score matching** | | | | | | | | | | | | | |
| --- | --- | --- | --- | --- | --- | --- | --- | --- | --- | --- | --- | --- | --- |
|  | | | Cohort | | | Patient count before matching | | | | | Patient count after matching | | |
|  | | | 1 – Pooled moderate- and high- immunosuppression | | | 34,429 | | | | | 16,328 | | |
|  | | | 2 – Low-immunosuppression | | | 18,963 | | | | | 16,328 | | |
| **Cohort 1 (N = 34,429) and cohort 2 (N = 18,963) characteristics before propensity score matching** | | | | | | | | | | | | | |
|  | **Demographics** | | | | | | | | | | | | |
|  |  | Cohort | | |  | Mean ± SD | Median | IQR | Patients | % of Cohort | | P-Value | Std diff. |
|  |  | 1 2 | | Age | Current Age | 49.2 +/- 12.2 57.8 +/- 11.5 | 49 59 | 19 17 | 34,429 18,963 | 100% 100% | | <0.001 | 0.727 |
|  |  | 1 2 | | AI | Age at Index | 41.6 +/- 11.1 47.5 +/- 10.9 | 41 49 | 17 16 | 34,429 18,963 | 100% 100% | | <0.001 | 0.532 |
|  |  | 1 2 | | 2054-5 | Black or African American |  |  |  | 5,627 2,558 | 16.3% 13.5% | | <0.001 | 0.080 |
|  |  | 1 2 | | M | Married |  |  |  | 7,376 4,292 | 21.4% 22.6% | | 0.001 | 0.029 |
|  |  | 1 2 | | 2106-3 | White |  |  |  | 24,011 14,172 | 69.7% 74.7% | | <0.001 | 0.112 |
|  |  | 1 2 | | 1002-5 | Native American or Alaska Native |  |  |  | 151 62 | 0.4% 0.3% | | 0.050 | 0.018 |
|  |  | 1 2 | | UNK | Unknown Race |  |  |  | 2,797 1,416 | 8.1% 7.5% | | 0.007 | 0.024 |
|  |  | 1 2 | | S | Never Married |  |  |  | 4,417 1,851 | 12.8% 9.8% | | <0.001 | 0.097 |
|  |  | 1 2 | | 2076-8 | Native Hawaiian or Other Pacific Islander |  |  |  | 38 13 | 0.1% 0.1% | | 0.134 | 0.014 |
|  |  | 1 2 | | UN | Unknown Ethnicity |  |  |  | 5,972 3,997 | 17.3% 21.1% | | <0.001 | 0.095 |
|  |  | 1 2 | | 2186-5 | Not Hispanic or Latino |  |  |  | 26,169 14,134 | 76.0% 74.5% | | <0.001 | 0.034 |
|  |  | 1 2 | | 2135-2 | Hispanic or Latino |  |  |  | 2,288 832 | 6.6% 4.4% | | <0.001 | 0.099 |
|  |  | 1 2 | | 2131-1 | Other Race |  |  |  | 1,451 616 | 4.2% 3.2% | | <0.001 | 0.051 |
|  |  | 1 2 | | 2028-9 | Asian |  |  |  | 354 126 | 1.0% 0.7% | | <0.001 | 0.040 |
|  | **Diagnosis** | | | | | | | | | | | | |
|  |  | Cohort | | |  | Mean ± SD | Median | IQR | Patients | % of Cohort | | P-Value | Std diff. |
|  |  | 1 2 | | Z72.0 | Tobacco use |  |  |  | 380 115 | 1.1% 0.6% | | <0.001 | 0.054 |
|  |  | 1 2 | | F17 | Nicotine dependence |  |  |  | 1,798 714 | 5.2% 3.8% | | <0.001 | 0.070 |
|  |  | 1 2 | | Z01.4 | Encounter for gynecological examination |  |  |  | 2,178 939 | 6.3% 5.0% | | <0.001 | 0.060 |
|  |  | 1 2 | | Z12.4 | Encounter for screening for malignant neoplasm of cervix |  |  |  | 1,669 702 | 4.8% 3.7% | | <0.001 | 0.057 |
|  |  | 1 2 | | Z55-Z65 | Persons with potential health hazards related to socioeconomic and psychosocial circumstances |  |  |  | 343 80 | 1.0% 0.4% | | <0.001 | 0.068 |
|  | **Medication** | | | | | | | | | | | | |
|  |  | Cohort | | |  | Mean ± SD | Median | IQR | Patients | % of Cohort | | P-Value | Std diff. |
|  |  | 1 2 | | 137 | HPV |  |  |  | 692 59 | 2.0% 0.3% | | <0.001 | 0.159 |
|  | **Laboratory** | | | | | | | | | | | | |
|  |  | Cohort | | |  | Mean ± SD | Median | IQR | Patients | % of Cohort | | P-Value | Std diff. |
|  |  | 1 2 | | 9083 | BMI | 30.1 +/- 8.2 30.0 +/- 7.9 | 28.620 28.622 | 11.058 10.410 | 11,963 4,657 | 34.7% 24.6% | | 0.562 | 0.010 |
| **Cohort 1 (N = 16,328) and cohort 2 (N = 16,328) characteristics after propensity score matching** | | | | | | | | | | | | | |
|  | **Demographics** | | | | | | | | | | | | |
|  |  | Cohort | | |  | Mean ± SD | Median | IQR | Patients | % of Cohort | | P-Value | Std diff. |
|  |  | 1 2 | | Age | Current Age | 55.8 +/- 10.7 56.0 +/- 11.1 | 57 57 | 16 17 | 16,328 16,328 | 100% 100% | | 0.107 | 0.018 |
|  |  | 1 2 | | AI | Age at Index | 45.9 +/- 10.5 46.2 +/- 10.9 | 47 47 | 16 17 | 16,328 16,328 | 100% 100% | | 0.019 | 0.026 |
|  |  | 1 2 | | 2054-5 | Black or African American |  |  |  | 2,271 2,280 | 13.9% 14.0% | | 0.886 | 0.002 |
|  |  | 1 2 | | M | Married |  |  |  | 3,860 3,757 | 23.6% 23.0% | | 0.178 | 0.015 |
|  |  | 1 2 | | 2106-3 | White |  |  |  | 12,195 12,084 | 74.7% 74.0% | | 0.160 | 0.016 |
|  |  | 1 2 | | 1002-5 | Native American or Alaska Native |  |  |  | 43 56 | 0.3% 0.3% | | 0.191 | 0.014 |
|  |  | 1 2 | | UNK | Unknown Race |  |  |  | 1,203 1,221 | 7.4% 7.5% | | 0.704 | 0.004 |
|  |  | 1 2 | | S | Never Married |  |  |  | 1,614 1,678 | 9.9% 10.3% | | 0.239 | 0.013 |
|  |  | 1 2 | | 2076-8 | Native Hawaiian or Other Pacific Islander |  |  |  | 12 12 | 0.1% 0.1% | | 1 | <0.001 |
|  |  | 1 2 | | UN | Unknown Ethnicity |  |  |  | 3,118 3,231 | 19.1% 19.8% | | 0.114 | 0.017 |
|  |  | 1 2 | | 2186-5 | Not Hispanic or Latino |  |  |  | 12,492 12,315 | 76.5% 75.4% | | 0.022 | 0.025 |
|  |  | 1 2 | | 2135-2 | Hispanic or Latino |  |  |  | 718 782 | 4.4% 4.8% | | 0.091 | 0.019 |
|  |  | 1 2 | | 2131-1 | Other Race |  |  |  | 491 561 | 3.0% 3.4% | | 0.028 | 0.024 |
|  |  | 1 2 | | 2028-9 | Asian |  |  |  | 113 114 | 0.7% 0.7% | | 0.947 | 0.001 |
|  | **Diagnosis** | | | | | | | | | | | | |
|  |  | Cohort | | |  | Mean ± SD | Median | IQR | Patients | % of Cohort | | P-Value | Std diff. |
|  |  | 1 2 | | Z72.0 | Tobacco use |  |  |  | 93 113 | 0.6% 0.7% | | 0.162 | 0.015 |
|  |  | 1 2 | | F17 | Nicotine dependence |  |  |  | 574 673 | 3.5% 4.1% | | 0.004 | 0.032 |
|  |  | 1 2 | | Z01.4 | Encounter for gynecological examination |  |  |  | 800 839 | 4.9% 5.1% | | 0.323 | 0.011 |
|  |  | 1 2 | | Z12.4 | Encounter for screening for malignant neoplasm of cervix |  |  |  | 566 632 | 3.5% 3.9% | | 0.052 | 0.022 |
|  |  | 1 2 | | Z55-Z65 | Persons with potential health hazards related to socioeconomic and psychosocial circumstances |  |  |  | 63 79 | 0.4% 0.5% | | 0.178 | 0.015 |
|  | **Medication** | | | | | | | | | | | | |
|  |  | Cohort | | |  | Mean ± SD | Median | IQR | Patients | % of Cohort | | P-Value | Std diff. |
|  |  | 1 2 | | 137 | HPV |  |  |  | 53 59 | 0.3% 0.4% | | 0.570 | 0.006 |
|  | **Laboratory** | | | | | | | | | | | | |
|  |  | Cohort | | |  | Mean ± SD | Median | IQR | Patients | % of Cohort | | P-Value | Std diff. |
|  |  | 1 2 | | 9083 | BMI | 29.6 +/- 8.0 30.1 +/- 8.0 | 28.190 28.715 | 10.738 10.644 | 4,351 4,351 | 26.6% 26.6% | | 0.006 | 0.059 |

Abbreviations: DMT, disease-modifying therapy; SD, standard deviation; std. diff,, standardized mean difference; HPV, human papilloma virus; BMI, body mass index; IQR, interquartile range.

**Supplementary Table 7. Baseline characteristics of cohorts of women with multiple sclerosis on high- versus moderate-immunosuppression, before and after propensity score matching.**

| **Cohort 1 and cohort 2 patient count before and after propensity score matching** | | | | | | | | | | | | | |
| --- | --- | --- | --- | --- | --- | --- | --- | --- | --- | --- | --- | --- | --- |
|  | | | Cohort | | | Patient count before matching | | | | | Patient count after matching | | |
|  | | | 1 – High immunosuppression | | | 14,917 | | | | | 9,737 | | |
|  | | | 2 – Moderate immunosuppression | | | 15,521 | | | | | 9,737 | | |
| **Cohort 1 (N = 14,917) and cohort 2 (N = 15,521) characteristics before propensity score matching** | | | | | | | | | | | | | |
|  | **Demographics** | | | | | | | | | | | | |
|  |  | Cohort | | |  | Mean ± SD | Median | IQR | Patients | % of Cohort | | P-Value | Std diff. |
|  |  | 1 2 | | Age | Current Age | 46.9 +/- 12.3 51.8 +/- 11.8 | 46 52 | 19 18 | 14,917 15,521 | 100% 100% | | <0.001 | 0.408 |
|  |  | 1 2 | | AI | Age at Index | 41.2 +/- 11.2 42.9 +/- 11.1 | 41 43 | 18 17 | 14,917 15,521 | 100% 100% | | <0.001 | 0.155 |
|  |  | 1 2 | | 2054-5 | Black or African American |  |  |  | 2,575 2,353 | 17.3% 15.2% | | <0.001 | 0.057 |
|  |  | 1 2 | | M | Married |  |  |  | 2,985 3,623 | 20.0% 23.3% | | <0.001 | 0.081 |
|  |  | 1 2 | | 2106-3 | White |  |  |  | 10,368 10,824 | 69.5% 69.7% | | 0.658 | 0.005 |
|  |  | 1 2 | | 1002-5 | Native American or Alaska Native |  |  |  | 72 56 | 0.5% 0.4% | | 0.100 | 0.019 |
|  |  | 1 2 | | UNK | Unknown Race |  |  |  | 947 1,597 | 6.3% 10.3% | | <0.001 | 0.143 |
|  |  | 1 2 | | S | Never Married |  |  |  | 1,910 2,094 | 12.8% 13.5% | | 0.076 | 0.020 |
|  |  | 1 2 | | 2076-8 | Native Hawaiian or Other Pacific Islander |  |  |  | 17 18 | 0.1% 0.1% | | 0.959 | 0.001 |
|  |  | 1 2 | | UN | Unknown Ethnicity |  |  |  | 2,377 3,075 | 15.9% 19.8% | | <0.001 | 0.101 |
|  |  | 1 2 | | 2186-5 | Not Hispanic or Latino |  |  |  | 11,356 11,594 | 76.1% 74.7% | | 0.004 | 0.033 |
|  |  | 1 2 | | 2135-2 | Hispanic or Latino |  |  |  | 1,184 852 | 7.9% 5.5% | | <0.001 | 0.098 |
|  |  | 1 2 | | 2131-1 | Other Race |  |  |  | 758 528 | 5.1% 3.4% | | <0.001 | 0.083 |
|  |  | 1 2 | | 2028-9 | Asian |  |  |  | 180 145 | 1.2% 0.9% | | 0.021 | 0.026 |
|  | **Diagnosis** | | | | | | | | | | | | |
|  |  | Cohort | | |  | Mean ± SD | Median | IQR | Patients | % of Cohort | | P-Value | Std diff. |
|  |  | 1 2 | | Z72.0 | Tobacco use |  |  |  | 216 134 | 1.4% 0.9% | | <0.001 | 0.055 |
|  |  | 1 2 | | F17 | Nicotine dependence |  |  |  | 920 642 | 6.2% 4.1% | | <0.001 | 0.092 |
|  |  | 1 2 | | Z01.4 | Encounter for gynecological examination |  |  |  | 1,101 852 | 7.4% 5.5% | | <0.001 | 0.077 |
|  |  | 1 2 | | Z12.4 | Encounter for screening for malignant neoplasm of cervix |  |  |  | 879 620 | 5.9% 4.0% | | <0.001 | 0.088 |
|  |  | 1 2 | | Z55-Z65 | Persons with potential health hazards related to socioeconomic and psychosocial circumstances |  |  |  | 207 105 | 1.4% 0.7% | | <0.001 | 0.070 |
|  | **Medication** | | | | | | | | | | | | |
|  |  | Cohort | | |  | Mean ± SD | Median | IQR | Patients | % of Cohort | | P-Value | Std diff. |
|  |  | 1 2 | | 137 | HPV |  |  |  | 430 162 | 2.9% 1.0% | | <0.001 | 0.133 |
|  | **Laboratory** | | | | | | | | | | | | |
|  |  | Cohort | | |  | Mean ± SD | Median | IQR | Patients | % of Cohort | | P-Value | Std diff. |
|  |  | 1 2 | | 9083 | BMI | 30.1 +/- 8.2 30.0 +/- 8.1 | 28.579 28.662 | 11.028 11.019 | 6,291 4,578 | 42.2% 29.5% | | 0.804 | 0.005 |
| **Cohort 1 (N = 9,737) and cohort 2 (N = 9,737) characteristics after propensity score matching** | | | | | | | | | | | | | |
|  | **Demographics** | | | | | | | | | | | | |
|  |  | Cohort | | |  | Mean ± SD | Median | IQR | Patients | % of Cohort | | P-Value | Std diff. |
|  |  | 1 2 | | Age | Current Age | 49.4 +/- 12.1 49.7 +/- 11.9 | 49 50 | 19 18 | 9,737 9,737 | 100% 100% | | 0.105 | 0.023 |
|  |  | 1 2 | | AI | Age at Index | 42.4 +/- 11.2 42.7 +/- 11.3 | 42 43 | 17 18 | 9,737 9,737 | 100% 100% | | 0.090 | 0.024 |
|  |  | 1 2 | | 2054-5 | Black or African American |  |  |  | 1,652 1,622 | 17.0% 16.7% | | 0.565 | 0.008 |
|  |  | 1 2 | | M | Married |  |  |  | 2,096 2,129 | 21.5% 21.9% | | 0.566 | 0.008 |
|  |  | 1 2 | | 2106-3 | White |  |  |  | 6,764 6,845 | 69.5% 70.3% | | 0.206 | 0.018 |
|  |  | 1 2 | | 1002-5 | Native American or Alaska Native |  |  |  | 39 38 | 0.4% 0.4% | | 0.909 | 0.002 |
|  |  | 1 2 | | UNK | Unknown Race |  |  |  | 759 709 | 7.8% 7.3% | | 0.175 | 0.019 |
|  |  | 1 2 | | S | Never Married |  |  |  | 1,313 1,270 | 13.5% 13.0% | | 0.364 | 0.013 |
|  |  | 1 2 | | 2076-8 | Native Hawaiian or Other Pacific Islander |  |  |  | 11 12 | 0.1% 0.1% | | 0.835 | 0.003 |
|  |  | 1 2 | | UN | Unknown Ethnicity |  |  |  | 1,667 1,658 | 17.1% 17.0% | | 0.864 | 0.002 |
|  |  | 1 2 | | 2186-5 | Not Hispanic or Latino |  |  |  | 7,449 7,474 | 76.5% 76.8% | | 0.672 | 0.006 |
|  |  | 1 2 | | 2135-2 | Hispanic or Latino |  |  |  | 621 605 | 6.4% 6.2% | | 0.637 | 0.007 |
|  |  | 1 2 | | 2131-1 | Other Race |  |  |  | 402 401 | 4.1% 4.1% | | 0.971 | 0.001 |
|  |  | 1 2 | | 2028-9 | Asian |  |  |  | 110 110 | 1.1% 1.1% | | 1 | <0.001 |
|  | **Diagnosis** | | | | | | | | | | | | |
|  |  | Cohort | | |  | Mean ± SD | Median | IQR | Patients | % of Cohort | | P-Value | Std diff. |
|  |  | 1 2 | | Z72.0 | Tobacco use |  |  |  | 126 116 | 1.3% 1.2% | | 0.518 | 0.009 |
|  |  | 1 2 | | F17 | Nicotine dependence |  |  |  | 522 491 | 5.4% 5.0% | | 0.317 | 0.014 |
|  |  | 1 2 | | Z01.4 | Encounter for gynecological examination |  |  |  | 610 608 | 6.3% 6.2% | | 0.953 | 0.001 |
|  |  | 1 2 | | Z12.4 | Encounter for screening for malignant neoplasm of cervix |  |  |  | 460 456 | 4.7% 4.7% | | 0.892 | 0.002 |
|  |  | 1 2 | | Z55-Z65 | Persons with potential health hazards related to socioeconomic and psychosocial circumstances |  |  |  | 104 89 | 1.1% 0.9% | | 0.278 | 0.016 |
|  | **Medication** | | | | | | | | | | | | |
|  |  | Cohort | | |  | Mean ± SD | Median | IQR | Patients | % of Cohort | | P-Value | Std diff. |
|  |  | 1 2 | | 137 | HPV |  |  |  | 149 154 | 1.5% 1.6% | | 0.772 | 0.004 |
|  | **Laboratory** | | | | | | | | | | | | |
|  |  | Cohort | | |  | Mean ± SD | Median | IQR | Patients | % of Cohort | | P-Value | Std diff. |
|  |  | 1 2 | | 9083 | BMI | 29.7 +/- 8.1 30.4 +/- 8.1 | 28.220 29.076 | 10.830 11.073 | 3,504 3,464 | 36.0% 35.6% | | <0.001 | 0.086 |

Abbreviations: DMT, disease-modifying therapy; SD, standard deviation; std. diff,, standardized mean difference; HPV, human papilloma virus; BMI, body mass index; IQR, interquartile range.
